# Supplementary figures and images for: Noninvasive vagus nerve stimulation alters neural response and physiological autonomic tone to noxious thermal challenge
Source: PLoS One. 2019 Feb 13;14(2):e0201212. doi: 10.1371/journal.pone.0201212 (PMC6373934; doi:10.1371/journal.pone.0201212)

**S1 Fig. nVNS versus sham numerical pain rating with maximal noxious thermal stimuli.**

**
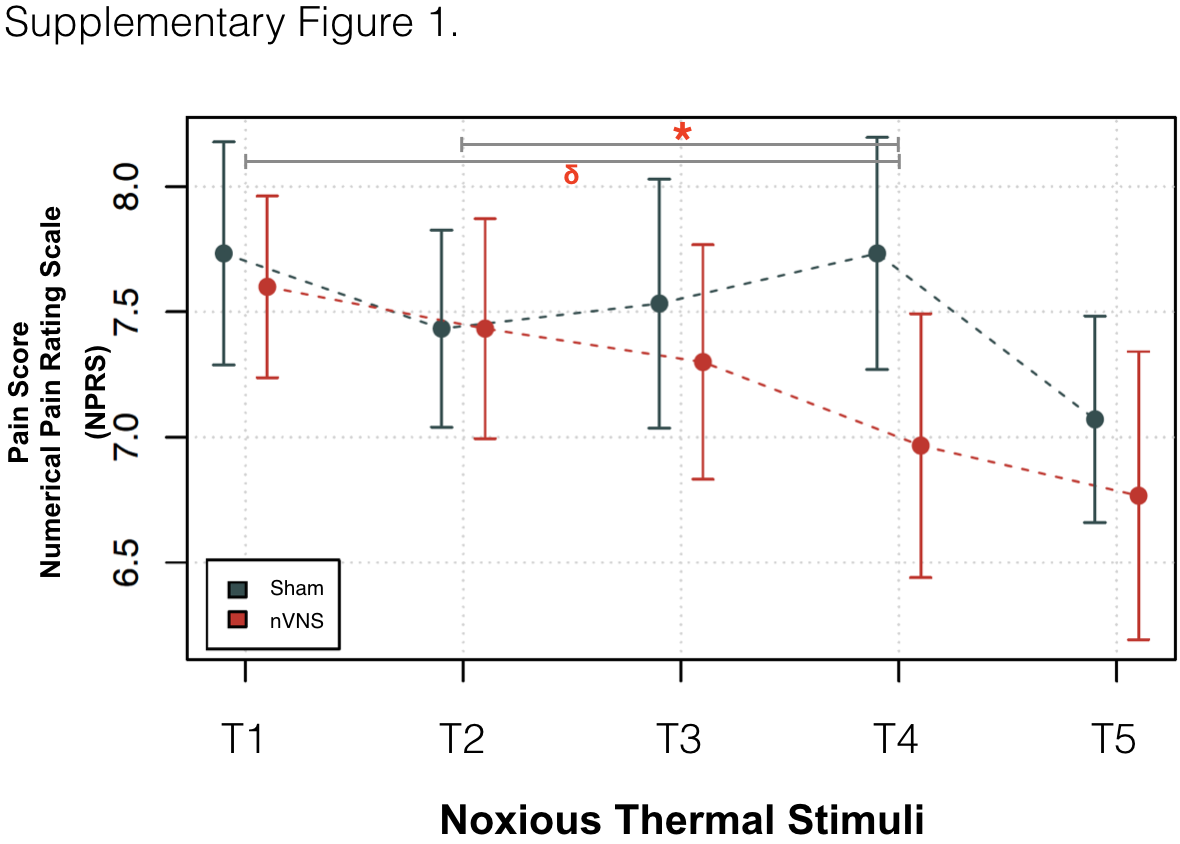
**

Supplement: S1 Fig — After either nVNS or sham stimulation, 5 successive noxious thermal stimuli were applied (up to 49.8°C) for 5 seconds each (T1-T5). Mean pain, as reported by subjects using the numerical pain rating scale (NPRS) after each noxious thermal stimulus did not differ between the sham and nVNS groups. Both groups had lower NPRS scores at T5 compared with T1 (NPRS decreased by -0.678 ± 0.209; t = -3.241; p = .002). In contrast to findings for the nVNS group, subjects who underwent sham stimulation had a positive slope in NPRS scores across thermal stimuli (i.e. the change in NPRS score with successive noxious thermal stimuli T1-T5) for T2 to T4 that was significantly different (slope in the sham group, 0.150 ± 0.122; vs the slope in the nVNS group, -0.233 ± 0.122; p = .0301) and also approached significance from T1 to T4 (sham group, 0.010 ± 0.847; vs nVNS group, -0.203 ± 0.847; p = .0785). Red circles = nVNS group. Blue circles = sham group. *p < .05; δp < .08. (DOCX) [file pone.0201212.s001.docx]
