# Supplementary material for: Noninvasive vagus nerve stimulation alters neural response and physiological autonomic tone to noxious thermal challenge
Source: PLoS One. 2019 Feb 13;14(2):e0201212. doi: 10.1371/journal.pone.0201212 (PMC6373934; doi:10.1371/journal.pone.0201212)
Supplement: S1 Table — The nVNS group showed significant decreases in the time to peak GSR for T1 and T2. There was no difference in GSR absolute mean change between groups for all time points examined. (DOCX) [file pone.0201212.s002.docx]

**S1 Table:**
