# Supplementary material for: Noninvasive vagus nerve stimulation alters neural response and physiological autonomic tone to noxious thermal challenge
Source: PLoS One. 2019 Feb 13;14(2):e0201212. doi: 10.1371/journal.pone.0201212 (PMC6373934; doi:10.1371/journal.pone.0201212)
Supplement: S2 Table — In the sham group, the time to peak GSR increased from T1 to T4 and T5. The mean GSR measured after the application of noxious thermal stimuli consistently increased from T2 to T5 and from T1 to T4. (DOCX) [file pone.0201212.s003.docx]

**S2 Table. Within-group comparisons for the time to peak GSR and absolute mean GSR for the sham stimulation group.**
