# Supplementary material for: Noninvasive vagus nerve stimulation alters neural response and physiological autonomic tone to noxious thermal challenge
Source: PLoS One. 2019 Feb 13;14(2):e0201212. doi: 10.1371/journal.pone.0201212 (PMC6373934; doi:10.1371/journal.pone.0201212)
Supplement: S3 Table — In the nVNS group, the time to peak GSR did not change between each successively applied noxious thermal stimulus. The mean GSR measured after each noxious thermal stimulus did not increase from T4 to T5, or from T1 to T2. (DOCX) [file pone.0201212.s004.docx]

**S3 Table. Within-group comparisons for the time to peak GSR and absolute mean GSR for the nVNS group.**
