# Supplementary material for: Noninvasive vagus nerve stimulation alters neural response and physiological autonomic tone to noxious thermal challenge
Source: PLoS One. 2019 Feb 13;14(2):e0201212. doi: 10.1371/journal.pone.0201212 (PMC6373934; doi:10.1371/journal.pone.0201212)
Supplement: S1 File — Heat tolerance and threshold measurements. Correlations between autonomic tone and pain reports. This file contains information on inclusion exclusion criteria, a description of heat tolerance and threshold measurements and finally pertinent correlations between autonomic tone and pain reports. (DOCX) [file pone.0201212.s005.docx]

**Supplementary File (S1 File)**

**Inclusion and exclusion criteria**

All subjects were instructed to refrain from taking any over-the-counter analgesics or anti-inflammatory medications, tobacco products, or alcoholic beverages for 1 week prior to the study visit. All subjects underwent phone screening up to 1 week prior to the fMRI scan. Subjects with prior surgery or abnormal anatomy at the treatment site (ie, anterior cervical neck region); injury; or abnormal anatomy at the MEDOC Peltier probe site [fMRI-compatible thermode, probe size 3 x 3 cm; TSA-II NeuroSensory Analyzer, MEDOC Advanced Medical Systems, Rimat Yishai, Israel), the left, lower leg anterior shin area; a history of neurologic disease (including transient ischemic attack, seizures, and syncope); a history of any type of implanted neurostimulator device, or cardiac pacemaker; or a history of cardiovascular disease or carotid artery disease were excluded from the study. Participants had no history of eating disorders and no current Diagnostic and Statistical Manual of Mental Disorders, 4^th^ edition (DSM-IV) Axis I psychiatric illnesses per self-report, or on standardized measures.

**Heat tolerance and threshold measurements**

Thermal thresholds and heat tolerance were calculated using the previously described method of limits (Yarnitsky & Sprecher, 1994) by taking the average of 5 thermal stimuli successively applied with a fMRI-compatible MEDOC probe to the right lower extremity (anterior shin) at an increasing slope of 1°C/s, from 32°C up to a maximum of 50°C. During MRI scan the temperature of 49.8°C was chosen based on heat tolerance obtained prior to the MRI scan. If tolerance was 50 degrees or above the temperature of 49.8 was chosen based on heat limitations provided by the MEDOC noxious thermal stimuli machine. For subjects with heat tolerance less than 49.8 (i.e., 44.7 °C) we used one degree higher than the tolerance reported (i.e., 45.7°C) to ensure maximal noxious stimulus was achieved.

**Correlations of interest**

**Correlations between autonomic tone and pain reports**

Autonomic measures taken during the application of thermal stimuli (time to peak GSR, mean GSR) and pain score were correlated within each group to better understand the relationships among these factors. In the nVNS group, mixed-model regression for the change in mean GSR with covariates for thermal stimuli showed significant negative correlations between mean GSR and thermal stimuli T3 (-0.955 ± 0.271; *t* = -3.528; *p* < .001), T4 (-1.429 ± 0.85; *t* = -5.106; *p* < .001), and T5 (-1.593 ± 0.295; *t* = -5.394; *p* < .001), that support the difference from baseline GSR to the peak slope (i.e., a decrease in slope from T3-T5 in the nVNS group only). Lastly, mixed-model regression results (for the nVNS group only) showed that the time to peak GSR covaried with thermal stimulus trial and pain score, with a significant effect of pain score detected (1.035 ± 0.449; *t* = 2.305; *p* = 0.025).
